# Supplementary figures and images for: Entamoeba histolytica extracellular vesicles drive pro-inflammatory monocyte signaling
Source: PLoS Negl Trop Dis. 2025 Apr 10;19(4):e0012997. doi: 10.1371/journal.pntd.0012997 (PMC12052212; doi:10.1371/journal.pntd.0012997)

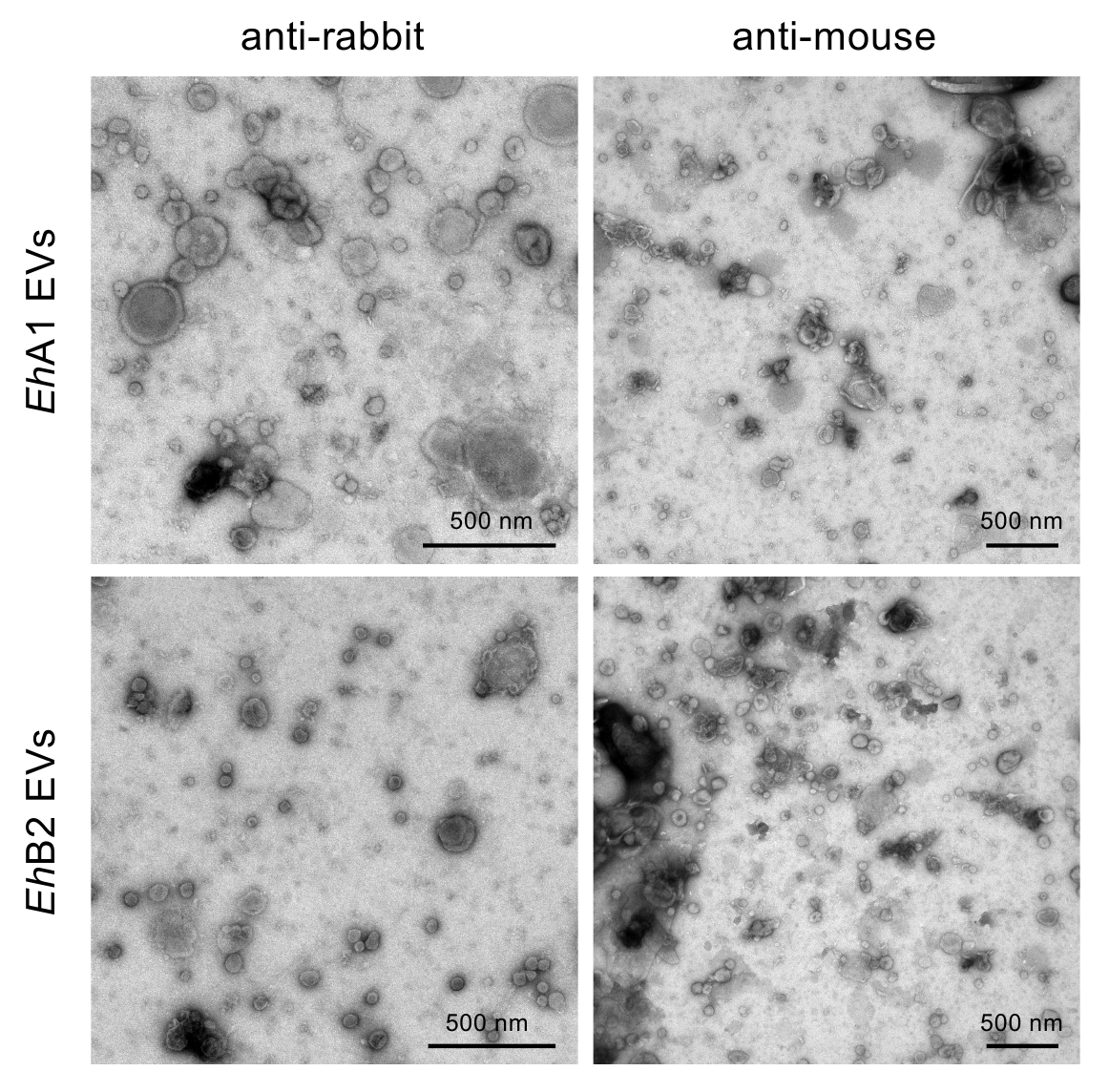

Supplement: S1 Fig — (TIF) [file pntd.0012997.s001.tif]

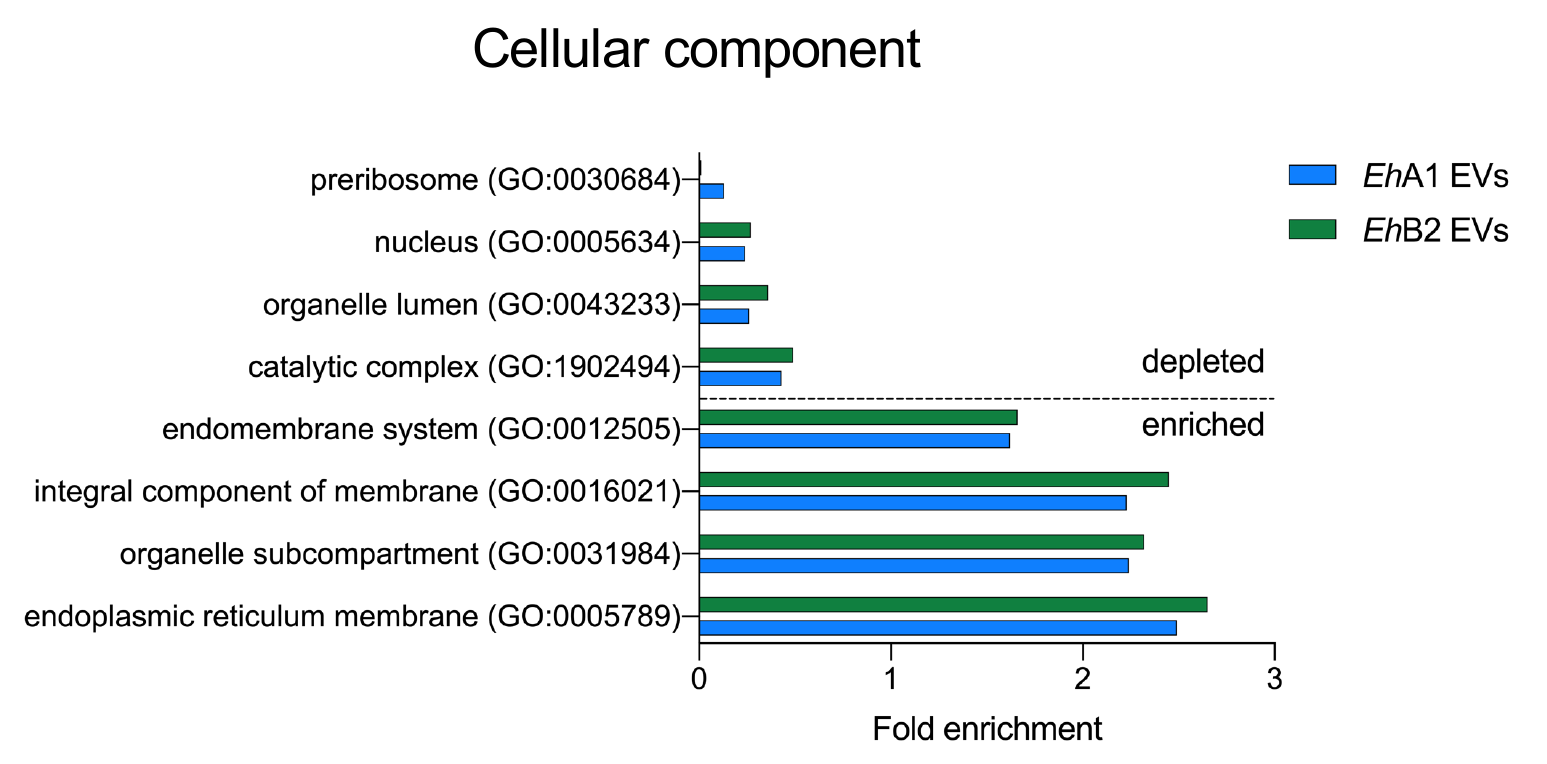

Supplement: S2 Fig — Shown are selected GO terms associated with proteins enriched or depleted in EV proteomes compared with trophozoite proteomes, based on statistical overrepresentation test performed with Panther knowledgebase [27,28]. (TIF) [file pntd.0012997.s002.tif]

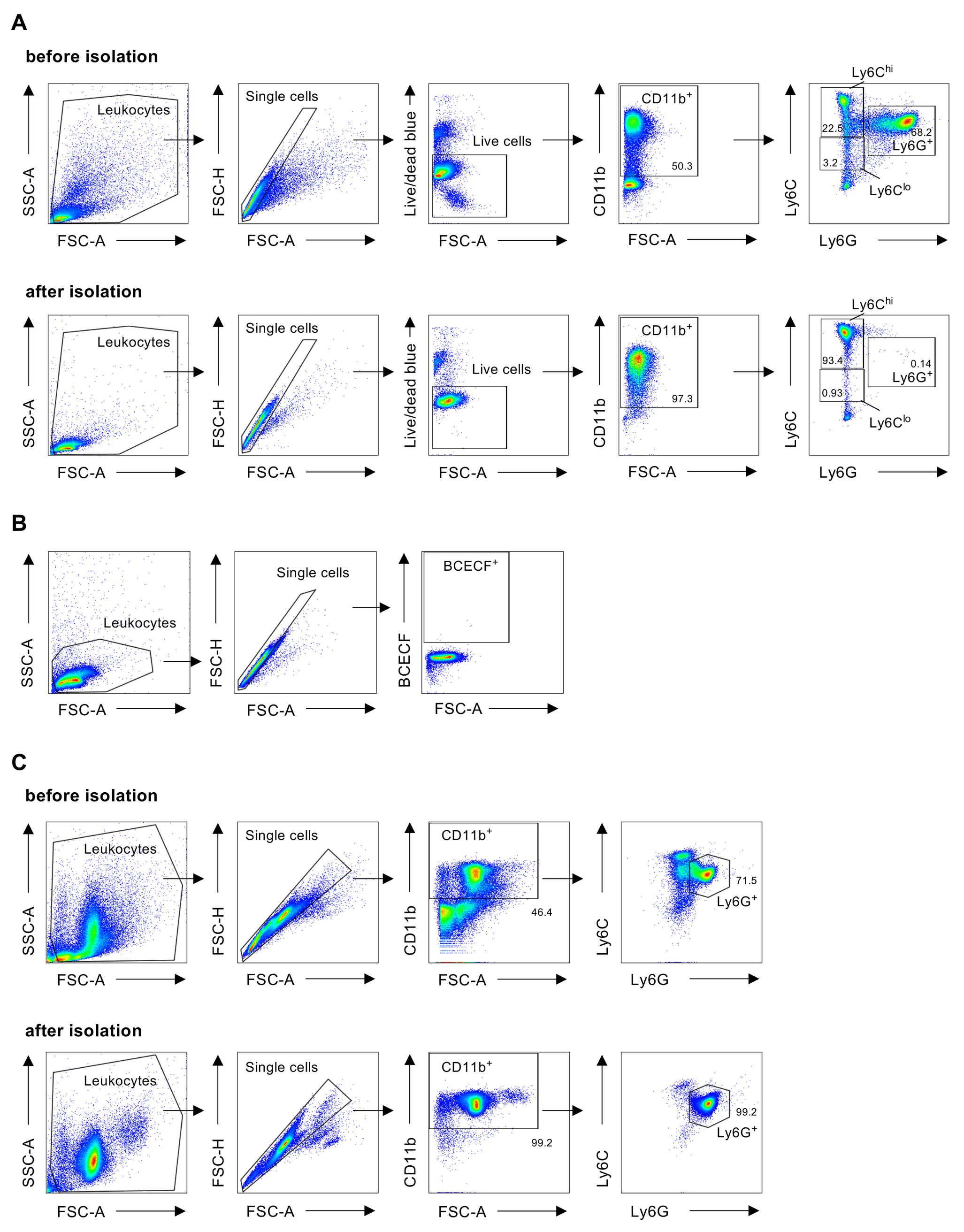

Supplement: S3 Fig — (A) Gating strategy used to control monocyte purity after isolation via flow cytometry. After gating for leukocytes, doublets were excluded by gating for FSC-A against FSC-H, followed by gating on live cells (live/dead blue-negative). Monocytes were identified as CD11b+Ly6C+Ly6G- cells and divided into cells expressing high amounts of Ly6C (Ly6Chi) or low amounts of Ly6C (Ly6Clo). Shown is a representative sample with a purity of 91.78% (CD11b+x(Ly6Chi+Ly6Clo)). (B) Gating strategy used on isolated monocytes to quantify uptake of BCECF-labeled EVs. (C) Gating strategy used to control neutrophil purity after isolation via flow cytometry. Neutrophils were identified as CD11b+Ly6C+Ly6G+ cells. Shown is a representative sample of bone marrow-derived neutrophils with a purity of 98.4% (CD11b+xLy6G+). (TIF) [file pntd.0012997.s003.tif]

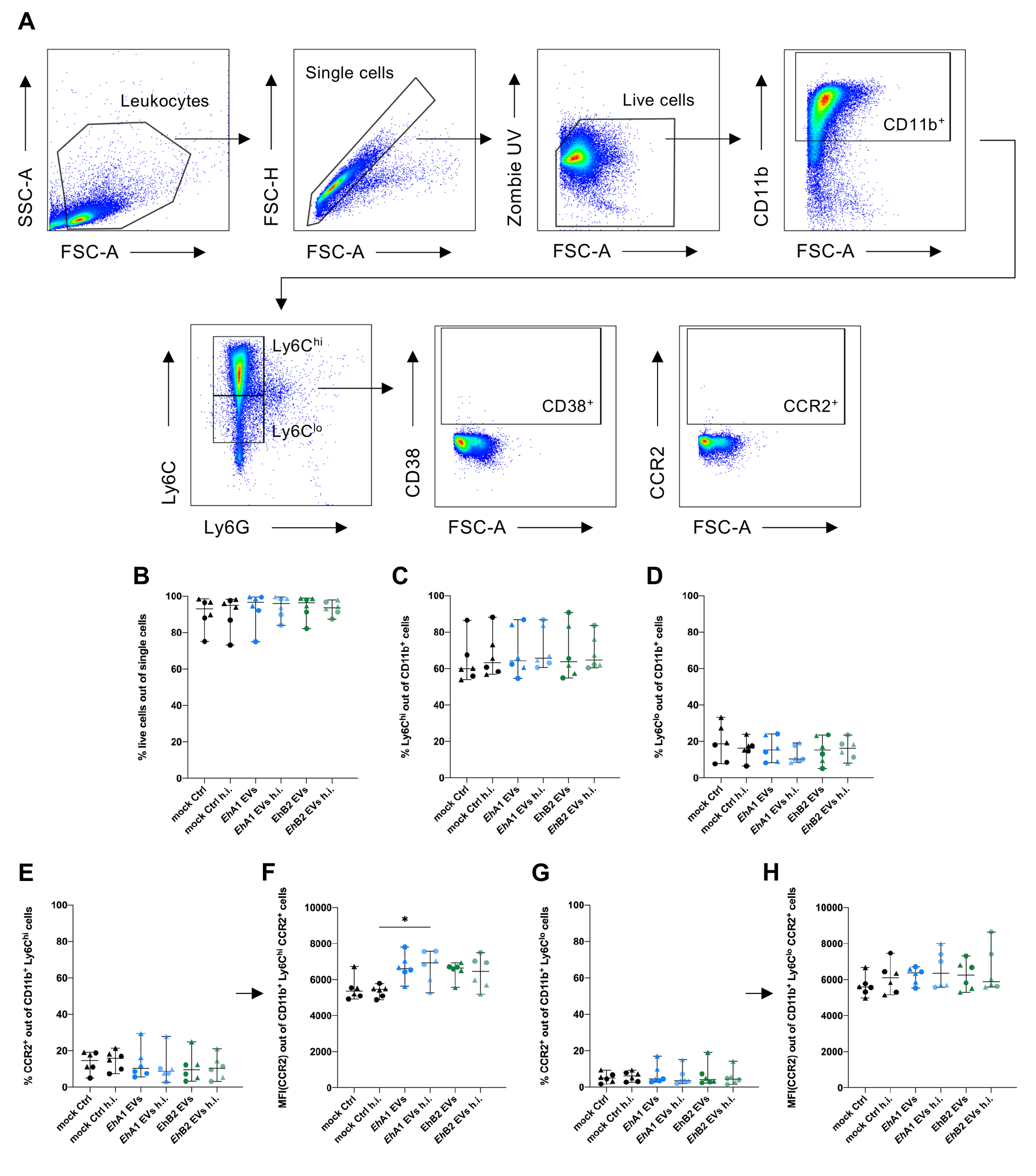

Supplement: S4 Fig — Bone marrow-derived monocytes of male (dots in graphs) and female (triangles in graphs) mice were stimulated for 24 h in vitro with 1000 EVs/cell or equal volume mock control and subsequently stained and analyzed by flow cytometry. To control for the effect of protein denaturation on stimulatory capacity, EV and control samples were heat inactivated (h.i.) at 95°C for 10 min prior to stimulation. (A) Gating strategy used to identify marker expression. After gating for leukocytes, doublets were excluded by gating for FSC-A against FSC-H, followed by gating on live cells (Zombie UV-negative). Monocytes were identified as CD11b+Ly6C+Ly6G- cells and divided into cells expressing high amounts of Ly6C (Ly6Chi) or low amounts of Ly6C (Ly6Clo). Expression of activation marker CD38 and chemokine receptor CCR2 was determined on both Ly6Chi and Ly6Clo monocytes. Gates were set according to fluorescence minus one controls, shown here for CD38 and CCR2. (B) Percent live cells following EV stimulation based on the Zombie UV versus FSC-A gate in (A). (C, D) Percent Ly6Chi (C) and Ly6Clo (D) monocytes out of CD11b+ cells. (E, F) Percent CCR2+ Ly6Chi monocytes (E) and median fluorescence intensity (MFI) of Ly6ChiCCR2+ cells (F) following EV stimulation. (G, H) Percent CCR2+ Ly6Clo monocytes (G) and median fluorescence intensity (MFI) of Ly6CloCCR2+ cells (H) following EV stimulation. (Kruskal-Wallis test with Dunn‘s multiple comparisons test, *p < 0.05, n = 6). (TIF) [file pntd.0012997.s004.tif]

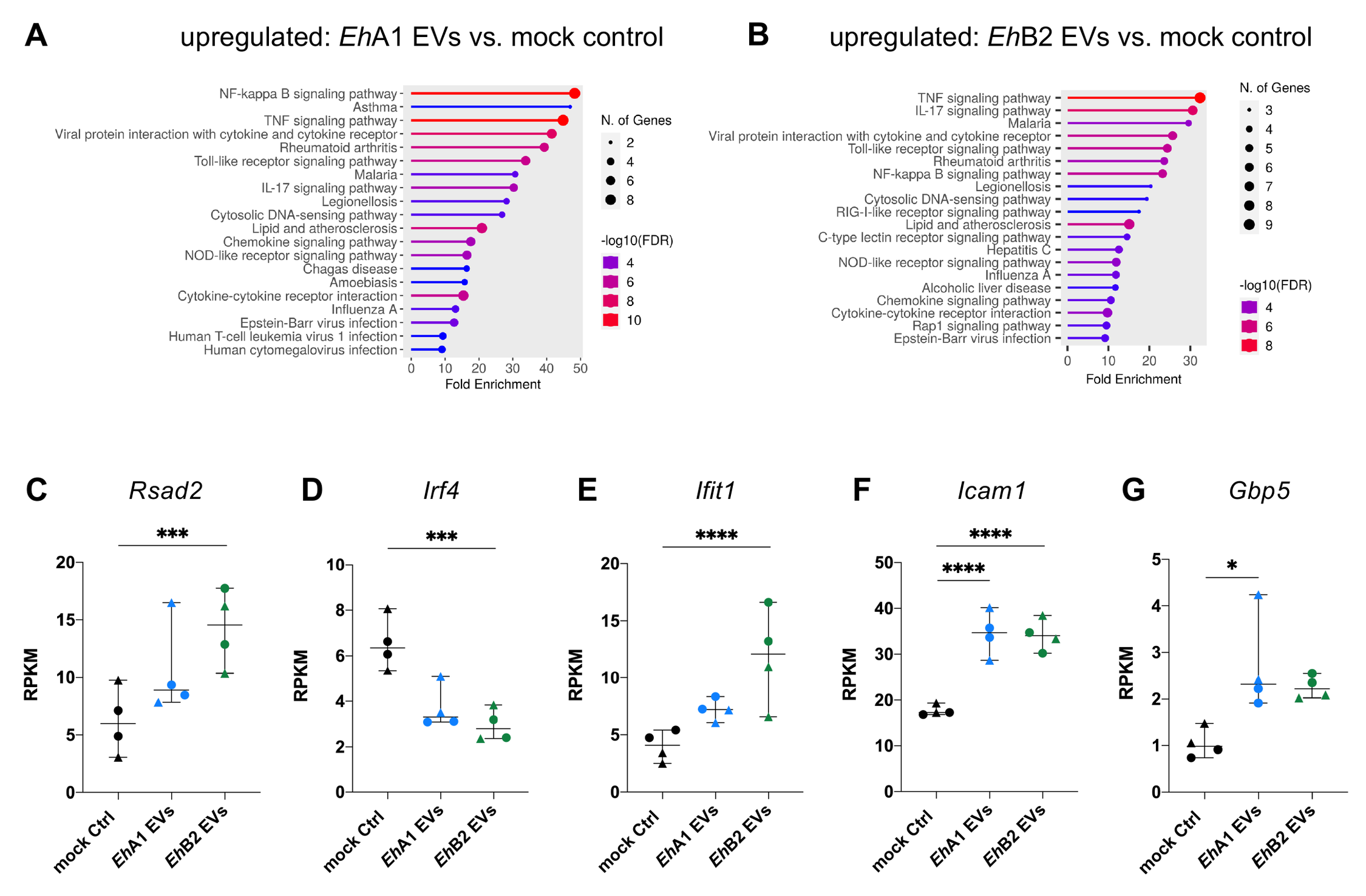

Supplement: S5 Fig — Monocytes isolated from bone marrow of male and female mice were stimulated for 8 h in vitro with 1000 EVs/cell or equal volume mock control. mRNA expression levels were subsequently analyzed using whole transcriptome sequencing. (A, B) KEGG pathway analysis of significantly upregulated genes in EhA1 EV (A) of EhB2 EV (B)-stimulated monocytes compared with mock controls (analysis performed with shinyGO version 80 [37], shown are the top 20 pathways). (C, D, E, F, G) Reads per kilobase per million mapped reads (RPKM) normalized expression values of selected interferon-stimulated genes in male- (dots) and female-derived (triangles) monocytes after EV stimulation compared with mock controls. (Bonferroni-corrected p value, *p < 0.05, *** p < 0.001, **** p < 0.0001, n = 4). (TIF) [file pntd.0012997.s005.tif]

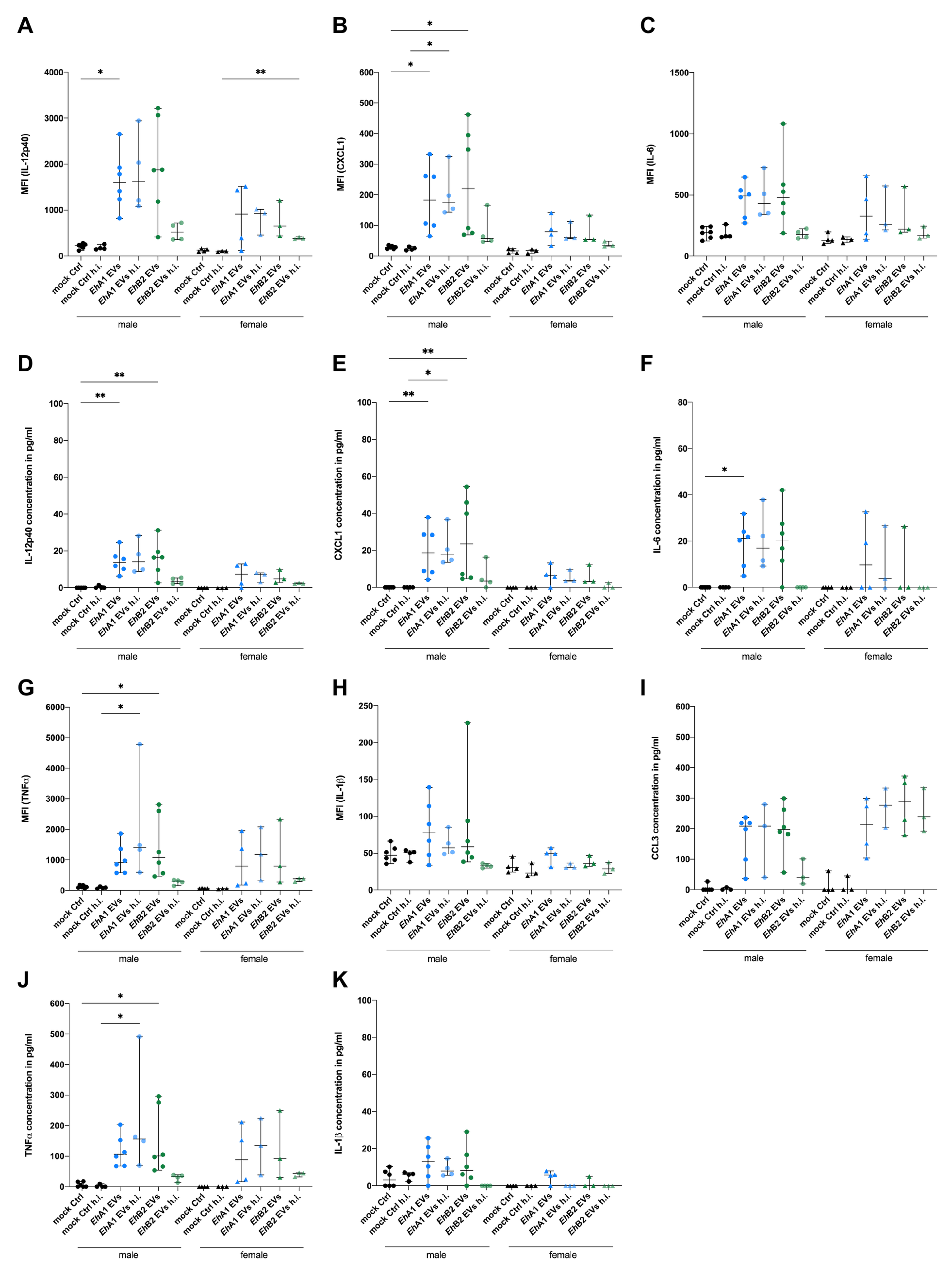

Supplement: S6 Fig — Monocytes were stimulated for 24 h in vitro with 1000 EVs/cell or equal volume mock control. Supernatants of stimulated cells were analyzed by ELISA or flow cytometry-based multiplex cytokine assay (LEGENDplex). To control for the effect of protein denaturation on stimulatory capacity, EV and control samples were heat inactivated (h.i.) at 95°C for 10 min prior to stimulation. (A–C, G, and H) Median fluorescence intensities (MFIs) for cytokines as determined by LEGENDplex and (D–F, J, and K) resulting calculated concentrations. (I) CCL3 concentration in supernatants as determined by ELISA. (One-way ANOVA with Dunnett‘s or Šídák‘s multiple comparisons test and Kruskal-Wallis test with Dunn‘s multiple comparisons test, * p < 0.05, ** p < 0.01, n = 3–6 (A–K)). (TIF) [file pntd.0012997.s006.tif]
