# Supplementary material for: Entamoeba histolytica extracellular vesicles drive pro-inflammatory monocyte signaling
Source: PLoS Negl Trop Dis. 2025 Apr 10;19(4):e0012997. doi: 10.1371/journal.pntd.0012997 (PMC12052212; doi:10.1371/journal.pntd.0012997)
Supplement: Attachment — Submitted filename: Honecker et al-Response to Reviewers.pdf [file pntd.0012997.s019.pdf]

Bernhard Nocht Institute for Tropical Medicine
P.O. Box 50 06 40, 22706 Hamburg, Germany

Anjan Debnath, Ph.D.
Laura-Isobel McCall, Ph.D.
Prof. Paul Brindley
Editors
PLOS Neglected Tropical Diseases

Dr. Barbara Honecker
Prof. Dr. Hanna Lotter
Prof. Dr. Iris Bruchhaus
RGs Molecular Infection Immunology
& Host-Parasite Interaction

Bernhard-Nocht-Str. 74
20359 Hamburg, Germany
phone +49 40 285380-475/472
barbara.honecker@bnitm.de
lotter@bnitm.de
bruchhaus@bnitm.de

Hamburg, 2025-02-27

Dear Editors,

Enclosed please find a revised version of our manuscript entitled "*Entamoeba histolytica* extracellular vesicles drive pro-inflammatory monocyte signaling" (PNTD-D-25-00034).

We thank the reviewers and the editorial team for the revision of our manuscript and the valuable comments. We appreciate the reviewers' satisfaction with our methodology and general study design. Regarding the Results and Discussion sections, we have addressed all reviewers' concerns and the according changes made to the manuscript in the point-by-point response below. Most importantly, as suggested by reviewers 1 and 3, we have significantly shortened the sections pertaining to the EV characterization, specifically the EV proteome, which we believe has improved readability and focus of our manuscript. As part of these changes, we have now removed the previous Figure S3, as we believe it did not contribute enough valuable information to the manuscript. Furthermore, we have made slight adjustments to Table S5, which has been uploaded in a new version.

All authors have approved the final manuscript. We thank you for your support and hope you will consider our manuscript for final publication in *PLOS Neglected Tropical Diseases*.

Yours sincerely,

B Honecker Dr. H. Lotter Iris Bruchhaus

Barbara Honecker, Hanna Lotter and Iris Bruchhaus

Reviewer's Comments

Results

Reviewer #1: The results presented in this article make an important contribution to understanding how the E. histolytica parasite communicates with the host's immune cells. A comprehensive omics approach has been used to characterize the content of E. histolytica EVs and their effect on monocyte activation markers. While the results are clearly presented, **some sections are dense; for instance, the proteomic comparisons could benefit from a more concise summary**. The figures are of high quality and effectively illustrate the key findings.

Response: We agree with the reviewer and have significantly shortened the proteome description from 3 to 2 pages in the Results section and from 2 to 1.5 pages in the Discussion, now focusing on the main points.

Reviewer #2: Their proteomic data shows that exosomes of the two Eh strains have expected proteins, as well as unique proteins. A strength here is that the exosome proteins are not contaminated by broken cells, and there are many differences between the strains. **A weakness here is that there are so many hypothetical proteins, which they should at least attempt to identify using AlphaFold and Foldseek. It is not clear how useful the miRNA data from exosomes, as there are so many targets in Eh and in host cells**. Transcript data from monocytes exposed to exosomes of virulent and avirulent Eh show expected increases in cytokines, which is greater in monocytes of male mice that have more severe liver disease. Further, the major finding of the paper, which was not expected, is that exosomes of avirulent EhA1 cause increased release of MPO in monocytes and neutrophils, which plays a protective role in Eh infection.

Response: We agree with the reviewer that the large amount of hypothetical, uncharacterized proteins weakens the interpretation of our data. As suggested, we have used AlphaFold with Foldseek to scan for similar proteins in other organisms that might hint at putative functions of hypothetical E. histolytica proteins. We have performed this analysis for 5 proteins of interest that were significantly different between EhA1 and EhB2 EV proteomes and have attached the findings below this response. While this strategy may help identifying putative functions or conserved domains for individual proteins, we noticed that most hits in other organisms correspond to more hypothetical and uncharacterized proteins. We thus believe that performing large-scale characterization for all hypothetical proteins detected in our EV proteomes using AlphaFold is outside the scope of our study and would likely not significantly enhance our data. However, we appreciate the suggestion and believe that this would be an interesting approach to increasing characterization of the E. histolytica proteome. Regarding the miRNA data, we are aware that, at the moment, it is difficult to ascertain the impact of individual miRNAs on target cells. Nevertheless, we believe that our target gene prediction provides an interesting first glimpse into possible EV-miRNA-mediated gene silencing in the host, that will need to be experimentally validated in further studies.

Reviewer #3: -Does the analysis presented match the analysis plan? Answer: No, because authors' aim is to study the role of EVs on male and female monocytes during the hepatic abscess formation due to E. histolytica infection and **an important part of the paper is focused to define the EV's content**. -Are the results clearly and completely presented? Answer: No, **the part corresponding to the EVs content is confuse and the link of the proteins and iRNA finding with monocytes activation is not clear**. -Are the figures (Tables, Images) of sufficient quality for clarity? Answer: Yes

Response: In our opinion, characterization of the EV content is important to gain insight into which components may be involved in causing the observed effects in monocytes to gain a bigger picture of EV-based communication. Nonetheless, as suggested also by Reviewer 1, we agree that the description of the EV content was too extensive and have now significantly shortened the corresponding sections in Results and Discussion, particularly the proteome part. Although we can not provide a direct link between protein and miRNA cargo and the observed effect on monocytes (as we now also stated more clearly in line 565 – 567), we interpreted our results in the context of previous studies and attribute our observations to the presence of several virulence factors (see also line 548 – line 565). We also speculate that the difference in susceptibility to heat inactivation of EhA1 and EhB2 EVs reveals an involvement of miRNAs in monocyte activation (line 601 – 608), which is an intriguing starting point for further studies in this field.

<u>Conclusions</u>

Reviewer #1: The conclusions are consistent with the presented data. However, while the discussion touches on experimental limitations, **it does not address the impact of EV isolation methods on the results** (see the following article as an example: https://doi.org/10.1016/j.jcyt.2023.11.001). Including a discussion on this matter would be a valuable addition to the manuscript.

Response: We thank the reviewer for this suggestion and agree that EV isolation is a crucial determinant in experimental outcome. In the context of our paper, we think this aspect is most important in the comparison of our EV proteome with that of two previous studies, which used different EV isolation methods, explaining some of the differences we observed. We now elaborated on this matter further (line 458 – 464) and highlighted the impact of the isolation method on EV cargo composition.

Reviewer #2: Yes, their conclusion that exosomes secreted by virulent Eh2B strain activate more cytokines in monocytes from male mice is supported by their data. Further they make the unexpected discovery that MPO is released by monocytes activated by exosomes of the less virulent Eh1A strain. In their lengthy discussion, **they do not talk enough abut the limitations of their results** (e.g., the absence of tight links between components of exosomes and responses of monocytes). **Nor do they concede the absence of experimental manipulation of either amoebae or the host**.

Response: We included further discussion on the limitations of our study, specifically the fact that we assessed EV composition right after EV isolation and not after co-culture with monocytes (line 565 – 567), and the limited comparability of our simple in vitro model to the complex interplay of cells and EVs in vivo (line 616 – 619). We also stated more clearly that, while we can assume that some virulence factors present in the EVs contribute to the observed effects in monocytes (as discussed in line 548 – 565), we can not directly link one with the other (line 565 – 567). We did not experimentally manipulate either amebae or host cells. Clones EhA1 and EhB2 were previously generated from HM-1:IMSS-A and -B cell lines by Meyer et al. (https://doi.org/10.1371/journal.ppat.1005853) and monocytes and neutrophils were isolated from wild type C57/BL6J mice as described in line 748 – 773. While it would be interesting to, for example, manipulate EVs to overexpress or silence certain markers or miRNAs to investigate their impact on monocyte stimulation more directly, we believe that this will need to be addressed in future studies.

Reviewer #3: -Are the conclusions supported by the data presented? Answer: Yes -Are the limitations of analysis clearly described? Answer: **No** -Do the authors discuss how these data can be helpful to advance our understanding of the topic under study? Answer: Yes and without any doubt the part corresponding to the monocytes activation is interesting and relevant for the field, but **the first part of the Results section is too long and the enormous number of molecules identified make lose the focus of the main aims**. -Is public health relevance addressed? Answer: Yes, amoebiasis continues to be a health problem in Mexico, India and other countries. It is necessary to continue studying the factors that cause its virulence to find a way to defeat it.

> *Response: As mentioned above in response to reviewer 2, we included further discussion on the limitations of our study, specifically the fact that we assessed EV composition right after EV isolation and not after co-culture with monocytes (line 565 – 567), and the limited comparability of our simple in vitro model to the complex interplay of cells and EVs in vivo (line 616 – 619). We also stated more clearly that, while we can assume that some virulence factors present in the EVs contribute to the observed effects in monocytes (as discussed in line 548 – 565), we can not directly link one with the other (line 565 – 567). Furthermore, we have significantly shortened the EV description in the Results section, specifically regarding the proteome and miRNA characterization, and believe that this has significantly improved readability.*

Editorial and Data Presentation Modifications

Reviewer #1: I strongly recommend summarizing the dense data sections, particularly in the proteomics part and the discussion, to improve readability.

> *Response: As stated above, we agree with the reviewer and have significantly shortened the proteome description from 3 to 2 pages in the Results section and from 2 to 1.5 pages in the Discussion, now focusing on the main points. We believe that this has significantly enhanced readability and focus of our manuscript.*

Reviewer #2: Is not clear whether they have deposited proteomic data to any accessible database. Further, it would be best to present peptide coverage for proteins in an Excel file, so one could judge relative abundance of proteins in exosomes.

> *Response: The raw proteomic data have been deposited to the ProteomeXchange Consortium via the PRIDE partner repository (https://www.ebi.ac.uk/pride/) with the dataset identifier PXD059387. Please also refer to the 'Data availability' section for more information on data repositories for the other sequencing data. Regarding peptide coverage, please refer to the excel files Table S1 (for EV proteomes) and Table S4 (for trophozoite proteomes), which should include all required information on the proteomes.*

Reviewer #3: Divide the paper in two. Leave in this all the related to the monocyte activation by EVs and the EVs characterization publish elsewhere.

> *Response: While we understand that there are two very different parts of our paper that might also be able to stand as papers on their own, we are convinced that the combination of both parts allows for the best interpretation of our results on monocyte activation. We believe that including the EV characterization is a valuable addition to the manuscript and, considering that neither of the other reviewers suggested to divide the paper, we believe that it is best to keep it as one. However, as mentioned above,*

we have now shortened the part describing the EV content, thus the focus of the paper has now shifted more towards the monocyte stimulation.

Summary and General Comments

Reviewer #1: The study is a well-conducted investigation into the role of E. histolytica EVs in immune modulation. Its strengths lie in its robust methodology and relevance to neglected tropical diseases. However, the **manuscript could benefit from improving the readability of the manuscript particularly in the proteomics part and the discussion**.

Response: We thank the reviewer for appreciating our methodology. As suggested and as mentioned above, we have re-written and shortened our Results and Discussion sections, particularly the proteome part, improving readability and focus.

Reviewer #2: This reviewer is not asking for new experiments, because an awful lot of work has already been done and three major results are clear. First, exosomes are different for avirulent versus virulent strains of Eh. Second, cytokine activation is also different for monocytes from male mice, which has worse disease, than from female mice. Third, exosomes from avirulent Eh induce an MPO response in monocytes that likely reduce liver pathology. Yes, the links between precise exosomal proteins and monocyte response are absent, but it is a good start.

Response: We thank the reviewer for acknowledging the amount of work that went into our study and agree that, while we can not directly link the EV cargo to the observed effects, our findings provide interesting insight for further studies.

Reviewer #3: In this paper authors propose to study the interaction of E. histolytica trophozoites with murine monocytes and its effect in hepatic abscesses formation. They also assessed myeloperoxidase released in neutrophils by extracellular vesicles stimulation. Authors have used two E. histolytica populations with different virulence to compare the effect and content of the EVs that these trophozoites secrete. The statement of the hypothesis and the strategy used are well supported and are adequate to resolve the scientific questions that the authors pose. Authors use to perform their study two E. histolytica clones with different virulence, their experiments are well performed with the adequate controls and correct statistical analysis of the results. There are studies supporting the fact that immune response in amoebiasis, mainly in liver infection has been documented by several authors, including Zhen et al and others. Thus, this manuscript will add new insights to the knowledge of the role of the immune response in the parasitic infection and it is relevant for public health. However, the manuscript addresses **two distinct issues**: n EVs content and role of male and female murine monocytes in infection. The section corresponding to EVs results cumbersome with many not sufficiently explained data. I **did not find a clear correlation between the enormous number of proteins and iRNA detected in vesicles with the main objective of the paper** that is to study the monocytes role in amoebiasis. **Which protein or proteins stimulate monocytes**? **What means the differences between EVs from pathogenic and non-pathogenic trophozoites**? In addition, to deep in the study ofn EVs, **authors should use the exosomes markers described for E. histolytica and other eukaryotic cells, such as Vps23**.

Response: In our opinion, including a comprehensive characterization of the EVs used for stimulation aids in understanding the observed effects on monocytes. For example, detection of virulence factors such as LPPG and Gal/GalNAc lectin, which are known to stimulate monocytes and macrophages through TLR2/4, in part explains the response detected in monocytes. For a more detailed discussion on the detected EV

components (mostly proteins) and their effect on monocytes, refer also to line 548 – line 565. Regarding the observed differences between EVs from pathogenic EhB2 and less pathogenic EhA1 amebae, these may aid in increasing our understanding of E. histolytica pathogenicity mechanisms, which are still poorly understood. The most intriguing finding of our study was the difference in the induction of MPO secretion, which corroborated the notion of a protective role of MPO in amebiasis, as suggested by another previous study. Therefore, analyzing EVs from both clones side by side provides interesting insight into protective and pathogenic mechanisms during E. histolytica infection.

Although we did not specifically mention EhVps23 (an ESCRT component) in our paper, we summarized proteins involved in EV biogenesis, specifically ESCRT proteins and tetraspanins, in Table S5, which also includes EhVps23. Markers typically used to characterize EVs in eukaryotes, including CD9, CD63 and CD81, are tetraspanins. We thus believe that, by providing proteome data of E.histolytica tetraspanins in our EVs, we present sufficient evidence for presence of EV markers. In addition, we compared our E. histolytica EV proteomes to the 100 most commonly identified EV proteins in eukaryotes and found many similarities, please refer also to Table S6.

Attachment, Response to Reviewer 2: AlphaFold Results

results based on AlphaFold with Foldseek Cluster, 25.02.2025
https://alphafold.ebi.ac.uk/

EHI_023070

AFDB accession	Description	Species	Sequence length	Average pLDDT ↑
AF-A0A662Y0L4-F1	GOLD domain-containing protein	*Nothophytophthora sp. Chile5*	200	89.5
AF-P0CN72-F1	Endoplasmic reticulum vesicle prote...	*Cryptococcus neoformans var. neofo...*	213	88.34
AF-A0A0N1PBE2-F1	Putative COP-coated vesicle membr...	*Leptomonas seymouri*	209	88
AF-V5ESK6-F1	GOLD domain-containing protein	*Kalmanozyma brasiliensis (strain GH...*	221	87.88
AF-A0A1Y2EZU7-F1	GOLD domain-containing protein	*Neocallimastix californiae*	216	87.31
AF-R9ALP7-F1	Endoplasmic reticulum vesicle prote...	*Wallemia ichthyophaga (strain EXF-...*	215	86.94
AF-A0A2N5V7L6-F1	GOLD domain-containing protein	*Puccinia coronata f. sp. avenae*	223	85.88
AF-A0A5B0NZN6-F1	Vesicle coat component	*Puccinia graminis f. sp. tritici*	223	85.62
AF-A0A0R3WW06-F1	GOLD domain-containing protein	*Hydatigena taeniaeformis*	208	85.44
AF-A0A507BTN9-F1	GOLD domain-containing protein	*Synchytrium microbalum*	212	85.44
AF-A0A0U1YP67-F1	ER vesicle protein	*Starmerella bombicola*	209	85.25
AF-A0A139AN08-F1	Endoplasmic reticulum vesicle prote...	*Gonapodya prolifera (strain JEL478)*	214	83.94
AF-A0A507ECB3-F1	GOLD domain-containing protein	*Powellomyces hirtus*	221	83.5
AF-A0A0L0SMD2-F1	GOLD domain-containing protein	*Allomyces macrogynus (strain ATCC ...*	230	83.31
AF-A0A6U3RJX4-F1	Hypothetical protein	*Ditylum brightwellii*	187	82.5
AF-A0A0H5RK26-F1	GOLD domain-containing protein	*Spongospora subterranea*	219	82.5
AF-A0A0G4GDD4-F1	GOLD domain-containing protein	*Chromera velia CCMP2878*	217	81.25
AF-A0A401PNW1-F1	GOLD domain-containing protein	*Scyliorhinus torazame*	171	78.94
AF-K2H3T1-F1	GOLD domain-containing protein	*Entamoeba nuttalli (strain P19)*	216	77.5
AF-A0A7R9G5L9-F1	Hypothetical protein	*Timema shepardi*	192	76.5
AF-J5SM45-F1	ER to golgi family transport-related ...	*Trichosporon asahii var. asahii (strai...*	202	76.44
AF-A0A0N5B988-F1	GOLD domain-containing protein	*Strongyloides papillosus*	177	74.06
AF-A0A1D2VD71-F1	GOLD domain-containing protein	*Ascoidea rubescens DSM 1968*	186	71.38
AF-A0A7S2MD41-F1	Hypothetical protein	*Haptolina brevifila*	193	67.81
AF-R7T3Y8-F1	GOLD domain-containing protein	*Capitella teleta*	184	60.97

EHI_130950

AFDB accession	Description	Species	Sequence length	Average pLDDT ↑
AF-F9F998-F1	Uncharacterized protein	*Fusarium oxysporum (strain Fo5176)*	273	72.62
AF-A0A6P7LFZ3-F1	gap junction Cx32.2 protein-like	*Betta splendens*	277	71.38
AF-A0A164QJN3-F1	Uncharacterized protein	*Sistotremastrum niveocremeum HHB...*	319	68.56
AF-A0A7S1C7Y3-F1	Hypothetical protein	*Bicosoecida sp. CB-2014*	284	68
AF-A0A164QJJ5-F1	Uncharacterized protein	*Sistotremastrum niveocremeum HHB...*	294	62.22
AF-B0EUM1-F1	Uncharacterized protein	*Entamoeba dispar (strain ATCC PRA-...*	255	57.34
AF-A0A2J6TAZ1-F1	Uncharacterized protein	*Hyaloscypha bicolor E*	269	54.34
AF-A0A5B0PH32-F1	Uncharacterized protein	*Puccinia graminis f. sp. tritici*	274	52.53
AF-A0A0R3S6K0-F1	Uncharacterized protein	*Elaeophora elaphi*	267	50.25

EHI_095850

AFDB accession	Description	Species	Sequence length	Average pLDDT ↑
AF-A0A5K1VCU5-F1	AAA_28 domain-containing protein	*Entamoeba histolytica*	240	88.56
AF-A0A2D4C0X5-F1	AAA_28 domain-containing protein	*Pythium insidiosum*	258	82.88
AF-I6TUE5-F1	AAA_28 domain-containing protein	*Encephalitozoon hellem (strain ATCC...*	245	82.06
AF-A0A4S8PM40-F1	AAA_28 domain-containing protein	*Glycomyces paridis*	278	74
AF-A0A7R8D4M1-F1	TRPL translocation defect protein 1...	*Lepeophtheirus salmonis*	234	72.62

EHI_072010

AFDB accession	Description	Species	Sequence length	Average pLDDT ↑
AF-A0A2P4S9T0-F1	Autophagy-related protein 9	*Bambusicola thoracicus*	472	91.62
AF-A0A1Y2F6T6-F1	Autophagy-related protein 9	*Neocallimastix californiae*	483	88.81
AF-A0A448WLN2-F1	Autophagy-related protein 9	*Protopolystoma xenopodis*	464	86.62
AF-A0A7S1VWH2-F1	Autophagy-related protein 9	*Grammatophora oceanica*	484	85.31
AF-A0A4P9WJL4-F1	Autophagy-related protein 9	*Blyttiomyces helicus*	457	84.75
AF-A0A1B7TB28-F1	Autophagy-related protein 9	*Hanseniaspora valbyensis NRRL Y-1...*	485	84.06
AF-A0A6C0EAF5-F1	Uncharacterized protein	*viral metagenome*	460	81.19
AF-I6ZKB7-F1	Autophagy-related protein 9	*Encephalitozoon romaleae (strain SJ...*	443	76.81
AF-M2RLC4-F1	Uncharacterized protein	*Entamoeba histolytica KU27*	535	73.69
AF-A0A7S1VS44-F1	Autophagy-related protein 9	*Sexangularia sp. CB-2014*	461	72.12
AF-B0EN77-F1	Autophagy-related protein 9	*Entamoeba dispar (strain ATCC PRA-...*	489	71.81
AF-B0EDX7-F1	Autophagy-related protein 9	*Entamoeba dispar (strain ATCC PRA-...*	533	70.81
AF-A0A0A1UB87-F1	Uncharacterized protein	*Entamoeba invadens IP1*	514	68.06
AF-A0A0A1UG60-F1	Autophagy-related protein 9	*Entamoeba invadens IP1*	548	55.91

EHI_161930

AFDB accession	Description	Species	Sequence length	Average pLDDT ↑
AF-A0A4R2JHB3-F1	Cation diffusion facilitator family tra...	*Blautia coccoides*	296	88.19
AF-B3RPP0-F1	G_PROTEIN_RECEP_F1_2 domain-c...	*Trichoplax adhaerens*	318	87.19
AF-A0A2H5YWE8-F1	Uncharacterized protein	*bacterium HR27*	280	86.19
AF-A0A7C6NAL9-F1	Cation diffusion facilitator family tra...	*Clostridiales bacterium*	278	86.12
AF-A0A6L3F2L6-F1	Uncharacterized protein	*Chloroflexi bacterium*	305	84.44
AF-M2RQ47-F1	Uncharacterized protein	*Entamoeba histolytica KU27*	256	83.94
AF-A0A2S6JD03-F1	Uncharacterized protein	*Microterricola pindariensis*	279	82.56
AF-A0A3D1ZB59-F1	Uncharacterized protein	*Dehalococcoidia bacterium*	297	81.38
AF-A0A452IGR5-F1	G_PROTEIN_RECEP_F1_2 domain-c...	*Gopherus agassizii (Agassiz's desert ...*	314	80.75
AF-A0A534WIR0-F1	Uncharacterized protein	*Deltaproteobacteria bacterium*	296	80.75
AF-W6L136-F1	Uncharacterized protein	*Phytomonas sp. isolate EM1*	266	80.44
AF-A0A1S1MMI6-F1	Uncharacterized protein	*Mycobacterium talmoniae*	286	79.88
AF-A0A3N5PYJ9-F1	Uncharacterized protein	*Chloroflexi bacterium*	310	79.81
AF-A0A7S4VTT7-F1	Hypothetical protein	*Alexandrium monilatum*	289	79.19
AF-A0A7C2D7X3-F1	Cation diffusion facilitator family tra...	*Bryobacterales bacterium*	281	77.38
AF-A0A522D040-F1	Cation transporter	*Alphaproteobacteria bacterium*	274	77.38
AF-K1R189-F1	Prostaglandin E2 receptor EP4 subt...	*Crassostrea gigas*	339	77.25
AF-A0A2A4EPU7-F1	Uncharacterized protein	*Pseudomonas acidophila*	279	76.62
AF-A0A060XE67-F1	G_PROTEIN_RECEP_F1_2 domain-c...	*Oncorhynchus mykiss*	335	76.62
AF-A0A846RR14-F1	Co/Zn/Cd efflux system component	*Arthrobacter pigmenti*	281	76.31
AF-S9UQK3-F1	Uncharacterized protein	*Strigomonas culicis*	284	74.62
AF-A0A6I4VT38-F1	Cation transporter	*Shimazuella sp. KC615*	275	74.25
AF-A0A1M5TZP5-F1	Uncharacterized protein	*Jatrophihabitans endophyticus*	273	74.06
AF-G0U7C5-F1	Uncharacterized protein	*Trypanosoma vivax (strain Y486)*	310	73.12
AF-A0A078HEA5-F1	BnaC02g38200D protein	*Brassica napus*	291	72.25
AF-M4DDS6-F1	Uncharacterized protein	*Brassica rapa subsp. pekinensis*	291	71.38
AF-A0A3L6GCV4-F1	BTB/POZ domain and ankyrin repeat...	*Zea mays*	318	68.31
AF-A0A7S4CZJ1-F1	Spindle pole body component	*Eutreptiella gymnastica*	292	67.62
AF-A0A433TFG9-F1	G_PROTEIN_RECEP_F1_2 domain-c...	*Elysia chlorotica*	368	66
AF-A0A488AGT0-F1	Cation efflux protein	*Clostridioides difficile*	296	65.94

EHI_161930 continued

AF-G3HF53-F1		KiSS-1 receptor	*Cricetulus griseus*	303	64.88
AF-M3UWR4-F1		Uncharacterized protein	*Entamoeba histolytica HM-1:IMSS-B*	283	64.12
AF-F4PXA1-F1		Uncharacterized protein	*Cavenderia fasciculata (strain SH3)*	330	63.25
AF-A0A0A1TZG3-F1		Uncharacterized protein	*Entamoeba invadens IP1*	285	62.59
AF-A0A6F9CK77-F1		G_PROTEIN_RECEP_F1_2 domain-c...	*Coregonus sp. 'balchen'*	277	60.81
AF-A0A542HAP0-F1		Uncharacterized protein	*Phycicoccus sp. SLBN-51*	260	57.41
AF-A0A7S4MJT2-F1		Hypothetical protein	*Odontella aurita*	295	50.28
AF-A0A540MFT1-F1		WAT1-related protein	*Malus baccata*	268	47.41
